# Bacterial Epidemiology and Antimicrobial Resistance in the Surgery Wards of a Large Teaching Hospital in Southern Italy

**DOI:** 10.4084/MJHID.2015.040

**Published:** 2015-06-01

**Authors:** Silvano Esposito, Renato Gioia, Giuseppe De Simone, Silvana Noviello, Domenico Lombardi, Vincenzo Giuseppe Di Crescenzo, Amelia Filippelli, Maria Rosaria Rega, Angelo Massari, Maria Giovanna Elberti, Lucilla Grisi, Giovanni Boccia, Francesco De Caro, Sebastiano Leone

**Affiliations:** 1 Division of Infectious Diseases, University of Salerno, Italy; 2 Division of Surgery, University of Salerno, Italy; 3 Division of Thoracic Surgery, University of Salerno, Italy; 4 Pharmacology Unit, University of Salerno, Italy; 5 Microbiology Unit, “San Giovanni di Dio e Ruggi d’Aragona Hospital”, Salerno, Italy; 6 Pharmacy Unit, “San Giovanni di Dio e Ruggi d’Aragona Hospital”, Salerno, Italy; 7 Hygiene Unit, University of Salerno, Italy

## Abstract

**Objectives:**

Surgical infections represent an increasingly important problem for the National Health System. In this study we retrospectively evaluated the bacterial epidemiology and antimicrobial susceptibility of the microorganisms concerned as well as the utilization of antibiotics in the General and Emergency Surgery wards of a large teaching hospital in southern Italy in the period 2011–2013.

**Methods:**

Data concerning non-duplicate bacterial isolates and antimicrobial susceptibility were retrieved from the Vitek 2 database. The pharmacy provided data about the consumption of antibiotics in the above reported wards. Chi-square or Fisher’s exact test were used.

**Results:**

In all, 94 Gram-negative were isolated in 2011, 77 in 2012, and 125 in 2013, *Escherichia coli, Acinetobacter baumannii* and *Pseudomonas aeruginosa* always being the most frequently isolated microorganisms. *A. baumannii* showed high rates of resistance to carbapenems (with values of 100% in 2011 and 2012) and low rates of resistance to tigecycline, colistin and amikacin. In the same years, there were respectively 105, 93, and 165 Gram-positive isolated. The rate of MRSA isolates ranged from 66% to 75% during the study period.

**Conclusions:**

Our results show no significant increase in antimicrobial resistance over the period in question, and a higher rate of both MRSA isolates and resistance to carbapenems in *A. baumannii* compared with other European data.

## Introduction

The considerable progress which occurred in the last century in treating post-operative infections led many authors to underestimate the problem of antibiotic resistance in the surgical field. This caused the re-emergence of the problem in the late 1990s and at the beginning of the new millennium, with a growing demand for multicenter studies that would contextualize the problem of either the epidemiology of these infections and the increasing antibiotic resistance or the increasingly innovative mechanisms put in place by pathogens to evade the action of antibiotics.^1^ Currently, the main problems related to antibiotic resistance consist in the increasing prevalence of extended spectrum beta-lactamases (ESBLs) producing *Enterobacteriaceae*, and in the increasing intra-hospital presence of methicillin-resistant *Staphylococcus aureus* (MRSA) and vancomycin-resistant enterococci (VRE), even though the spread of multi-drug resistant (MDR) bacteria greatly differs according to the geographic areas.^2^–^5^ Knowledge of the local epidemiology of antibiotic resistance may help to develop therapeutic strategies and stewardship of more effective measures to optimize therapeutic outcomes and reduce the length of hospital stay.^6^–^9^ In this study we retrospectively examined the bacterial epidemiology of the major Gram-negative and Gram-positive pathogens isolated in the Departments of General Surgery and Emergency Surgery of the Salerno University Hospital “San Giovanni di Dio e Ruggi d’Aragona” in the period 2011–2013, paying special attention to the antibacterial susceptibility profile of *Klebsiella pneumoniae, Pseudomonas aeruginosa*, *Acinetobacter baumannii* and *Staphylococcus aureus*.

## Materials and Methods

Data concerning non-duplicate Gram-positive and Gram-negative microorganisms isolated from different clinical specimens of patients hospitalized in the period 2011–2013 at the General Surgery and Emergency Surgery of “San Giovanni di Dio e Ruggi d’Aragona”, a university hospital having 900 bed capacity with major programs in a broad range of clinical specialties located in Salerno (Italy), were retrieved from the database of the Laboratory of Bacteriology of the same hospital. Overall, the number and type of admission to the hospital did not change over time in the study period. In these wards, mainly patients undergoing major elective and urgent/emergency abdominal procedures are admitted. Microbial isolates are labelled as clinically relevant (both community- and healthcare-acquired) according to the information as detailed in the laboratory request form. We also focused on the evaluation of any change over time of the main expressions of resistance, with particular regard to the microorganisms considered to be the most difficult to treat in surgical wards, *i.e., K. pneumoniae, A. baumannii*, *P. aeruginosa* and *S. aureus*. In particular, we analyzed rates of resistance to carbapenems (imipenem, meropenem, and ertapenem), amikacin, tigecycline, and colistin in the Gram-negative and of methicillin-resistance in *S. aureus*. Microbial identification and antibiotic susceptibility testing were performed by the fully automated system VITEK 2 (bioMérieux, Marcy l’Etoile, France). Colleagues at the Department of Pharmacy provided data on consumption, in the years 2011–2013, of antibiotics with a spectrum of activity mainly directed against Gram-negative microorganisms, *i.e.*, imipenem, meropenem, ertapenem, amikacin, tigecycline, and colistin. Antibiotic usage was evaluated using defined daily doses (DDDs). Indeed, in our study, we defined the consumption of drugs as DDD/100 bed days.^10^ Chi-square or Fisher’s exact test were used to analyze possible significant differences in isolation rate. Statistical significance was established at a two-tailed level of <5 %.

## Results

Overall, 296 Gram-negative bacteria were isolated in the two departments considered in the period 2011–2013: 94 in 2011, 77 in 2012, and 125 in 2013. The three most represented pathogens were, in descending order, *E. coli, A. baumannii,* and *P. aeruginosa*. This order was constantly maintained throughout the three years, as well as the presence of other microorganisms such as *K. pneumoniae* and *Proteus* spp (Table 1). A total of 363 Gram-positive bacteria were isolated in the three-year period: 105 in 2011, 93 in 2012, and 165 in 2013. Table 2 shows the frequencies of isolation of Gram-positive microorganisms isolated from 2011 to 2013, and the trend did not significantly change over the study period. The resistance rates of the main Gram-negative microorganisms in the Departments of General Surgery and Emergency Surgery during the period 2011–2013 are reported in Table 3. *A. baumannii* showed high rates of resistance to carbapenems (with values of 100% in 2011 and 2012) and low rates of resistance to tigecycline, colistin, and amikacin. *P. aeruginosa* showed variable resistance rates, between 11 and 60%, to carbapenems (imipenem and meropenem) and amikacin, whereas all isolates proved to be susceptible to colistin. *K. pneumoniae* showed variable resistance rates to carbapenems and tigecycline, whereas all isolates were susceptible to colistin and 2 of the through 2011 were resistant to amikacin. Of note, the prevalence of isolation of *Klebsiella* producing carbapenemase was higher in intensive care units than surgical wards in the study period. Resistance rates of the main Gram-positive microorganisms are described in Table 4. The rate of MRSA isolates ranged from 66% to 75% during the study period. Moreover, no *S. aureus* isolate proved to be resistant to daptomycin, linezolid, tigecycline, and glycopeptides. Table 5 shows the antibiotics usage rates of surgical wards expressed by DDD/100 bed days.

## Discussion

Bacterial infections in surgical wards are an increasing problem.^11^–^13^ It has been estimated that in Italy surgical infections represent about 14% of all nosocomial infections, a rate lower than in Europe where the incidence is around 20%.^14^ In recent years, considerable importance has also been given to their impact on health care costs. For example, a patient with a post-surgical infection can cost the National Health System up to 325 for a single day’s hospitalization, with a high risk of re-hospitalization when the antibiotic therapy is inappropriate.^3^ The largest Italian study published in 2009 primarily aimed to investigate the epidemiology of the microorganisms responsible for infections in surgical wards, and the modifiable risk factors showed that *E. coli, E. faecalis*, and *S. aureus* are the most common pathogens responsible for surgical site infections (SSIs).^3^ In particular, an increased rate of SSIs due to MRSA was reported, with a mortality rate of 5%. Data concerning the increasing rate of SSIs sustained by MRSA were in agreement with those of the study by Manian that relates such an increase to a number of factors: age over 70 years, surgery duration over 4 hours, and hospital stay longer than 3 days.^6^ In the present study, we report a high rate of MRSA (60–80% of all *S. aureus* isolates) in the three years. These values are in agreement with those reported by Shree (60% of MRSA isolates).^15^ Methicillin resistance was only based on phenotypic resistance (Vitek 2) and was not confirmed neither by detection of PBP2a by latex agglutination tests nor by molecular detection of the *mecA* gene. While this can represent a limit of our study, it must be said that in a study by Nonhoff et al., Vitek 2 system detected oxacillin resistance with a sensitivity/specificity of 99/96%.

In Europe, the MYSTIC (Meropenem Yearly Susceptibility Test Information Collection) study has turned its attention to ESBL-producing bacteria. In particular, it was observed that the expression of ESBL differs according to the region considered, with higher values being observed in Italy, Poland, and Russia. Another interesting consideration comes from MYSTIC data concerning SSIs sustained by *P. aeruginosa*: isolation of *P. aeruginosa* in the course of SSIs does not necessarily involve its etiological role. The excessive use of carbapenems also employed for their anti-pseudomonas spectrum might have resulted in an increase in the resistance of these pathogens to this class of antibiotics.^17^ These same data are confirmed by the results of our study where the resistance of *P. aeruginosa* to carbapenems lies between 40 and 60%. This datum is in agreement with those of the European surveillance program of infections EARS-Net (Annual Report of the European Antimicrobial Resistance Surveillance Network) which reports, regard to Italy, a *P. aeruginosa* resistance to these antibiotics more than 50% in 2012.^18^

In Asia, however, the SMART study showed, between 2008 and 2009, a positive trend in the isolation of ESBL-producing enterobacteria in the intra-abdominal infections (IAIs), with rates of 10% in Australia, Japan, Korea and Singapore, and 50% in India, China and Thailand.^19^ The same study also showed that ESBL production is closely related to increased mortality in the IAIs in agreement with data reported previously by Esposito.^20^ The rise in mortality from surgical infections was attributed to inadequate antimicrobial treatment defined as the lack of administration of antibiotics for an infection or administration of antibiotics to which the microorganism responsible for the infection is resistant.^20^ In our study, we focused on the following Gram-negative pathogens: *A. baumannii, P. aeruginosa,* which, along with *E. faecium* and *S. aureus*, mainly “escape” the effects of antibiotics due to MDR mechanisms.^21^–^25^ Our results related to *A. baumannii* are in agreement with those of the study on the SENTRY Antimicrobial Surveillance Program (2006–2009) reported by Gales with the exception of a higher resistance to colistin (values between 4.7 and 8.3% versus 0.9% in the SENTRY study).^26^ The resistance rate to tigecycline for this microorganism was not tested in the SENTRY study. However, our data (15–25%) are in agreement with the findings (24%) of Fernandez-Cuenca.^27^ Concerning the antibiotic resistance pattern of *P. aeruginosa* and *K. pneumoniae*, we compared our data with those from the EARS-Net study. The resistance of *P. aeruginosa* to carbapenems detected in our study (30–60%) was similar to that provided by EARS-Net (25–50%), whereas resistance to amikacin (38–40%) was much greater than that reported in the above study (10–25%).^18^ Resistance to colistin was not tested in the EARS-Net study; however, our data (0% resistance) differ from those reported by the CANWARD study (6.9% resistance).^28^ The resistance of *K. pneumoniae* to carbapenems that we detected (16–50%) is in agreement with that provided by EARS-Net (25–50%). While the resistance to colistin and amikacin was not tested in the EARS-Net study. Our data (0% resistance to both) are similar to those reported by the CANWARD study (0% resistance to colistin and amikacin at 0.4%).^28^ Our data, albeit limited due to the small number of *K. pneumoniae* isolates, are in agreement with findings elsewhere that report increasing antibiotic resistance of this microorganism to carbapenems and colistin.^29^,^30^ During our observation period, carbapenems (especially meropenem and imipenem) were found to be the most frequently used antibiotics. In addition, we observed an increasing use over the years of tigecycline that can be explained by the rise of surgical infections sustained by strains of ESBL-producing bacteria, for which tigecycline proves to be an effective antibiotic.^17^ The same considerations are also appropriate for amikacin and colistin whose increasing use, according to Shree, could be explained by their effectiveness for the treatment of infections caused by ESBL-producing strains.^15^ In conclusion, it may be stated that present findings, together with a previous one,^31^–^34^ have significant clinical implications in fighting infections caused by MDR bacteria in general surgery and emergency surgery wards. Indeed, through better understanding of the local bacterial epidemiology, it is possible to obtain useful information to administer an appropriate empiric therapy and prevent the spread of antibiotic resistant organisms.

## Figures and Tables

**Table 1:** Epidemiology of Gram-negative bacteria in the Departments of General Surgery and Emergency Surgery in the period 2011–2013*

Microorganism	2011 No. (%)	2012 No. (%)	2013 No. (%)
*Escherichia coli*	30 (31.9)	31 (40.3)	37 (29.6)
*Acinetobacter baumannii*	21 (22.3)	12 (15.6)	20 (16.0)
*Pseudomonas aeruginosa*	14 (14.9)	9 (11.7)	15 (12.0)
*Proteus spp*	7 (7.4)	-	9 (7.2)
*Klebsiella pneumoniae*	10 (10.6)	6 (7.8)	18 (14.4)
*Morganella morganii*	-	5 (6.5)	1 (0.8)
*Enterobacter* spp.	3 (3.2)	4 (5.2)	5 (4.0)
*Klebsiella oxytoca*	2 (2.1)	1 (1.3)	5 (4.0)
Others**	7 (7.4)	9 (11.7)	15 (12.0)
Total	94 (100)	77 (100)	125 (100)

* No significant differences in isolation rate were observed.

** *Bacteroides fragilis* (2), *Citrobacter freundii* (6), *Hafnia alvei* (2), *Klebsiella ozaenae* (1), *Stenotrophomonas maltophilia* (1), *Citrobacter braakii* (2), *Haemophilus parainfluenzae* (2), *Pantoea* spp (1), *Salmonella* spp (2), *Serratia marcescens* (2), *Veillonella* spp (1), *Achromobacter xylosidans* (1), *Acinetobacter lwoffii* (1), *Acinetobacter ursingii* (1), *Bacteroides stercoris* (1), *Bacteroides thetaiotaomicron* (1), *Citrobacter koseri* (2), *Providencia stuartii* (1), *Shewanella algae* (1).

**Table 2:** Epidemiology of Gram-positive bacteria in the Departments of General Surgery and Emergency Surgery in the period 2011–2013*

Micrororganism	2011 No. (%)	2012 No. (%)	2013 No. (%)
*Enterococcus faecalis*	31 (29.5)	26 (28.0)	36 (21.8)
CoNS°	22 (21.0)	14 (15.1)	34 (20.6)
*Staphylococcus epidermidis*	14 (13.3)	14 (15.1)	32 (19.4)
*Enterococcus faecium*	14 (13.3)	15 (16.1)	18 (10.9)
*Staphylococcus aureus*	12 (11.4)	14 (15.1)	12 (7.3)
*Enterococcus avium*	2 (1.9)	-	6 (3.6)
Others**	10 (9.5)	10 (10.8)	27 (16.4)
*Total*	105 (100)	93 (100)	165 (100)

* No significant differences in isolation rate were observed.

** *Streptococcus agalactiae* (4)*, Streptococcus galloticus spp. pasteurianus* (1)*, Streptococcus gordonii* (2)*, Streptococcus mitis* (2)*, Streptococcus parasanguinis* (3)*, Streptococcus sanguinis* (2)*, Actynomices naeslundii* (1)*, Corynebacterium striatum* (2)*, Granulicatella adiacens* (1)*, Streptococcus dysgalactiae* (1)*, Streptococcus intermedius* (1)*, Enterococcus caselliflavus* (1)*, Enterococcus durans* (1)*, Enterococcus gallinarum* (2)*, Enterococcus hirae* (2)*, Enterococcus raffinosus* (6)*, Gemella morbillorum* (1)*, Leuconostoc speudomesenteroides* (1)*, Pediococcus pentosaceus* (1)*, Propionibacterium acnes* (1)*, Stenotrophomonas maltophilia* (1)*, Streptococcus anginosus* (6)*, Streptococcus constellatus* (1)*, Streptococcus equinus* (1)*, Streptococcus salivarius* (2)*.* °CoNS :coagulase-negative staphylococci other than *Staphylococcus epidermidis*

**Table 3:** Rates of resistance of the main Gram-negative microorganisms in the Departments of General Surgery and Emergency Surgery in the period 2011–2013.

	2011			2012			2013		
	KP (n=10)	AB (n=21)	PA (n=14)	KP (n=6)	AB (n=12)	PA (n=9)	KP (n=18)	AB (n=20)	PA (n=15)
MEM	3 (30%)	21 (100%)	6 (46.2%)	3 (50%)	12 (100%)	1 (11%)	4 (22.2%)	13 (65%)	6 (40%)
IPM	0 (0%)	21 (100%)	4 (30.8%)	0 (0%)	12 (100%)	1 (11%)	3 (16.7%)	10 (50%)	9 (60%)
ETP	3 (30%)	21 (100%)	-	0 (0%)	12 (100%)	-	5 (27.8%)	20 (100%)	-
TGC	0 (0%)	3 (14.3%)	-	0 (0%)	3 (25 %)	-	6 (33.3%)	0 (0%)	-
AMK	2 (20%)	0 (0%)	5 (38.5%)	0 (0%)	0 (0%)	1 (11%)	0 (0 %)	0 (0%)	6 (40%)
CST	0 (0%)	1 (4.8%)	0 (0%)	0 (0%)	1 (8.3%)	0 (0%)	0 (0%)	0 (0%)	0 (0%)

Legend: KP: *K pneumoniae*; AB: A*. baumannii*; PA: *P. aeruginosa*; MEM: meropenem, IPM: imipenem; ETP: ertapenem; TGC: tigecycline; AMK: amikacin; CST: colistin.

**Table 4:** Rates of resistance of the main Gram-positive microorganisms in the Departments of General Surgery and Emergency Surgery in the period 2011–2013.

	2011				2012				2013			
	SA (n=12)	SE (n=14)	EFs (n=31)	EFm (n=14)	SA (n=14)	SE (n=14)	EFs (n=26)	EFm (n=15)	SA (n=12)	SE (n= 32)	EFs (n=36)	EFm (n=18)
**OXA**	9 (75%)	12 (85.7%)	-	-	10 (71.4%)	13 (92.8%)	-	-	8 (66.7%)	26 (81.2%)	-	-
**DAP**	0 (0%)	0 (0%)	NA	NA	0 (0%)	0 (0%)	NA	NA	0 (0%)	0 (0%)	NA	NA
**LNZ**	0 (0%)	0 (0%)	0 (0%)	0 (0%)	0 (0%)	0 (0%)	0 (0%)	0 (0%)	0 (0%)	0 (0%)	0 (0%)	0 (0%)
**TEC**	0 (0%)	0 (0%)	0 (0%)	0 (0%)	0 (0%)	4 (28.6%)	0 (0%)	0 (0%)	0 (0%)	12 (37.5%)	0 (0%)	0 (0%)
**TGC**	0 (0%)	0 (0%)	0 (0%)	0 (0%)	0 (0%)	0 (0%)	0 (0%)	0 (0%)	0 (0%)	0 (0%)	0 (0%)	0 (0%)
**VAN**	0 (0%)	0 (0%)	0 (0%)	0 (0%)	0 (0%)	0 (0%)	0 (0%)	0 (0%)	0 (0%)	0 (0%)	0 (0%)	3 (16.7%)

Legend: SA: *Staphylococcus. aureus*; SE: *Staphylococcus epidermidis*; EFs: *Enterococcus faecalis*; EFm: *Enterococcus faecium*; NA: not available; OXA: oxacillin; DAP: daptomycin; LNZ: linezolid; TEC: teicoplanin; TGC: tigecycline; VAN: vancomycin.

**Table 5:** Antibiotics usage rates in the Departments of General Surgery and Emergency Surgery in the period 2011–2013.

		2011	2012	2013
	ATC/DDD/Unit	DDD/100 bed days	DDD/100 bed days	DDD/100 bed days
**MEM**	J01DH02/2/g	38,5	40.4	36.9
**IPM**	J01DH51/2/g	0.39	2.7	7.8
**ETP**	J01DH03/1/g	16.9	8.6	14.3
**TGC**	J01AA12/0.1/g	6	5.2	14.0
**AMK**	J01GB06/1/g	-	1.03	14.3
**CST**	J01XB01/3/MU	3.1	2.6	8.8

Legend: MEM: meropenem, IPM: imipenem; ETP: ertapenem; TGC: tigecycline; AMK: amikacin; CST: colistin.

## References

[1] Ramcharan AA, den Heijer CD, Smeets EE, Rouflart MM, van Tiel FH, Bruggeman CA, Breukink SO, Tordoir JH, Baeten CG, Stobberingh EE (2014). Microbiology of surgical site infections after gastrointestinal surgery in the south region of The Netherlands. Future Microbiol.

[2] Montravers P, Lepape A, Dubreuil L, Gauzit R, Pean Y, Benchimol D, Dupont H (2009). Clinical and microbiological profiles of community-acquired and nosocomial intra-abdominal infections: results of the French prospective, observational EBIIA study. J Antimicrob Chemother.

[3] De Werra C, Schiavone D, Di Micco R, Triassi M (2009). Surgical site infections in Italy. Infez Med.

[4] Stefani S (2009). Evolution in the antibiotic susceptibility and resistance. Infez Med.

[5] Esposito S, Capuano A, Noviello S, Mazzeo F, Ianniello F, Filippelli A, Rossi F, Leone S (2003). Modification of patients’ endogenous bacterial flora during hospitalization in a large teaching hospital in Naples. J Chemother.

[6] Manian FA, Meyer PL, Setzer J, Senkel D (2003). Surgical site infections associated with methicillin-resistant Staphylococcus aureus: do postoperative factors play a role?. Clin Infect Dis.

[7] Esposito S, Ianniello F, Leone S, Noviello S, Marvaso A, Iannantuoni N, Esposito E, Imperato L, Aiello D, Aloisio T, Maio P, Acierno D, Romano G, Patrelli G (2003). Multicentre survey of post-surgical infections in Campania (Italy). Infez Med.

[8] Drago L (2007). Epidemiology and mechanisms of resistance: clinical and environmental impact. Infez Med.

[9] Camporese A, Santini G (1999). Surveillance of antibiotic-resistant microorganisms for the rational use of antimicrobial drugs. Infez Med.

[10] WHO Collaborating Centre for Drug Statistics Methodology http://www.whocc.no/atc_ddd_index/.

[11] Owens CD, Stoessel K (2008). Surgical site infections: epidemiology, microbiology and prevention. J Hosp Infect.

[12] Leaper DJ (2010). Risk factors for and epidemiology of surgical site infections. Surg Infect.

[13] Young MH, Washer L, Malani PN (2008). Surgical site infections in older adults: epidemiology and management strategies. Drugs Aging.

[14] Esposito S, Leone S, Noviello S, Lanniello F, Fiore M (2007). Antibiotic resistance in long-term care facilities. New Microbiol.

[15] Shree N, Arora BS, Mohil RS, Kasana D, Biswal I (2013). Bacterial profile and patterns of antimicrobial drug resistance in intra-abdominal infections: current experience in a teaching hospital. Indian J Pathol Microbiol.

[16] Nonhoff C, Rottiers S, Struelens MJ (2005). Evaluation of the Vitek 2 system for identification and antimicrobial susceptibility testing of Staphylococcus spp. Clin Microbiol Infect.

[17] Nicoletti G, Nicolosi D, Rossolini GM, Stefani S (2008). Etiology, epidemiology and microbiological diagnosis of intra-abdominal infections. Infez Med.

[18] European Centre for Disease Prevention and Control (2012). Antimicrobial resistance surveillance in Europe 2011. Annual Report of the European Antimicrobial Resistance Surveillance Network (EARS-Net).

[19] Sheng WH, Badal RE, Hsueh PR, SMART Program (2013). Distribution of extended-spectrum β-lactamases, AmpC β-lactamases, and carbapenemases among Enterobacteriaceae isolates causing intra-abdominal infections in the Asia-Pacific region: results of the study for Monitoring Antimicrobial Resistance Trends (SMART). Antimicrob Agents Chemother.

[20] Esposito S, Leone S, Carosi G (2009). Analysis of current guidelines for intra-abdominal infections. J Chemother.

[21] Deveci Ö, Dal T, Tekin R, Bozkurt F, Tekin A, Dayan S (2013). Carbapenem resistance in Acinetobacter baumannii: where is it heading?. Infez Med.

[22] Bassetti M (2007). Strategies for management of difficult to treat Gram-negative infections: focus on Pseudomonas aeruginosa. Infez Med.

[23] Ece G, Samlioglu P, Atalay S, Kose S (2014). Evaluation of the in vitro colistin susceptibility of Pseudomonas aeruginosa and Acinetobacter baumannii strains at a tertiary care centre in Western Turkey. Infez Med.

[24] Rice LB (2008). Federal funding for the study of antimicrobial resistance in nosocomial pathogens: no ESKAPE. J Infect Dis.

[25] Ippolito G, Leone S, Lauria FN, Nicastri E, Wenzel RP (2010). Methicillin-resistant Staphylococcus aureus: the superbug. Int J Infect Dis.

[26] Gales AC, Jones RN, Sader HS (2011). Contemporary activity of colistin and polymyxin B against a worldwide collection of Gram-negative pathogens: results from the SENTRY Antimicrobial Surveillance Program (2006–09). J Antimicrob Chemother.

[27] Fernández-Cuenca F, Tomás-Carmona M, Caballero-Moyano F, Bou G, Martínez-Martínez L, Vila J, Pachón J, Cisneros JM, Rodríguez-Ba-o J, Pascual A (2013). In vitro activity of 18 antimicrobial agents against clinical isolates of Acinetobacter spp.: multicenter national study GEIH-REIPI-Ab 2010. Enferm Infecc Microbiol Clin.

[28] Karlowsky JA, Adam HJ, Baxter MR, Lagacé-Wiens PR, Walkty AJ, Hoban DJ, Zhanel GG (2013). Antimicrobial susceptibility of 22746 pathogens from Canadian hospitals: results of the CANWARD 2007–11 study. J Antimicrob Chemother.

[29] Morrissey I, Hackel M, Badal R, Bouchillon S, Hawser S, Biedenbach D (2013). A Review of Ten Years of the Study for Monitoring Antimicrobial Resistance Trends (SMART) from 2002 to 2011. Pharmaceuticals (Basel).

[30] Castanheira M, Mendes RE, Woosley LN, Jones RN (2011). Trends in carbapenemase-producing Escherichia coli and Klebsiella spp. from Europe and the Americas: report from the SENTRY antimicrobial surveillance programme (2007–09). J Antimicrob Chemother.

[31] Manfredi R, Nanetti A (2009). An active microbiological surveillance project at an Italian teaching hospital: microbial isolates, recent epidemiological trends, major clinical concerns, and antimicrobial susceptibility rates during a four-year period. Infez Med.

[32] Leone S, Stefani S, Venditti M, Grossi P, Colizza S, De Gasperi A, Scaglione F, Sganga G, Esposito S (2011). Intra-abdominal infections: model of antibiotic stewardship in an era with limited antimicrobial options. Int J Antimicrob Agents.

[33] Esposito S, Leone S, Noviello S, Ianniello F (2004). Management of severe bacterial infections and role of the infectious disease specialist: results of an interview-based survey. Infez Med.

[34] Esposito S, Leone S, Noviello S (2005). Management of severe bacterial infections. Expert Rev Anti Infect Ther.

